# Antiviral Activity of Repurposing Ivermectin against a Panel of 30 Clinical SARS-CoV-2 Strains Belonging to 14 Variants

**DOI:** 10.3390/ph15040445

**Published:** 2022-04-02

**Authors:** Océane Delandre, Mathieu Gendrot, Priscilla Jardot, Marion Le Bideau, Manon Boxberger, Céline Boschi, Isabelle Fonta, Joel Mosnier, Sébastien Hutter, Anthony Levasseur, Bernard La Scola, Bruno Pradines

**Affiliations:** 1 Unité Parasitologie et Entomologie, Département Microbiologie et Maladies Infectieuses, Institut de Recherche Biomédicale des Armées, 13005 Marseille, France; o.delandre@gmail.com (O.D.); ma.gendrot@laposte.net (M.G.); isabelle.fonta.09@gmail.com (I.F.); joelmosnier@orange.fr (J.M.); 2 Aix Marseille University, IRD, SSA, AP-HM, VITROME, 13005 Marseille, France; sebastien.hutter@univ-amu.fr; 3 IHU Méditerranée Infection, 13005 Marseille, France; priscilla.jardot@univ-amu.fr (P.J.); marion.le-bideau@ap-hm.fr (M.L.B.); manon.boxberger@hotmail.fr (M.B.); celine.boschi@ap-hm.fr (C.B.); anthony.levasseur@univ-amu.fr (A.L.); bernard.la-scola@univ-amu.fr (B.L.S.); 4 Aix Marseille University, IRD, AP-HM, MEPHI, 13005 Marseille, France; 5 Centre National de Référence du Paludisme, 13005 Marseille, France

**Keywords:** COVID-19, SARS-CoV-2, in vitro, ivermectin, remdesivir, chloroquine, repurposing drug, omicron

## Abstract

Over the past two years, several variants of SARS-CoV-2 have emerged and spread all over the world. However, infectivity, clinical severity, re-infection, virulence, transmissibility, vaccine responses and escape, and epidemiological aspects have differed between SARS-CoV-2 variants. Currently, very few treatments are recommended against SARS-CoV-2. Identification of effective drugs among repurposing FDA-approved drugs is a rapid, efficient and low-cost strategy against SARS-CoV-2. One of those drugs is ivermectin. Ivermectin is an antihelminthic agent that previously showed in vitro effects against a SARS-CoV-2 isolate (Australia/VI01/2020 isolate) with an IC_50_ of around 2 µM. We evaluated the in vitro activity of ivermectin on Vero E6 cells infected with 30 clinically isolated SARS-CoV-2 strains belonging to 14 different variants, and particularly 17 strains belonging to six variants of concern (VOC) (variants related to Wuhan, alpha, beta, gamma, delta and omicron). The in vitro activity of ivermectin was compared to those of chloroquine and remdesivir. Unlike chloroquine (EC_50_ from 4.3 ± 2.5 to 29.3 ± 5.2 µM) or remdesivir (EC_50_ from 0.4 ± 0.3 to 25.2 ± 9.4 µM), ivermectin showed a relatively homogeneous in vitro activity against SARS-CoV-2 regardless of the strains or variants (EC_50_ from 5.1 ± 0.5 to 6.7 ± 0.4 µM), except for one omicron strain (EC_50_ = 1.3 ± 0.5 µM). Ivermectin (No. EC_50_ = 219, mean EC_50_ = 5.7 ± 1.0 µM) was, overall, more potent in vitro than chloroquine (No. EC_50_ = 214, mean EC_50_ = 16.1 ± 9.0 µM) (*p* = 1.3 × 10^−34^) and remdesivir (No. EC_50_ = 201, mean EC_50_ = 11.9 ± 10.0 µM) (*p* = 1.6 × 10^−13^). These results should be interpreted with caution regarding the potential use of ivermectin in SARS-CoV-2-infected patients: it is difficult to translate in vitro study results into actual clinical treatment in patients.

## 1. Introduction

In December 2019, a new disease called coronavirus disease 2019 (COVID-19), caused by severe acute respiratory syndrome coronavirus (SARS-CoV-2), began to spread all over the world [1]. Over the past two years, several emerging variants of SARS-CoV-2 have been detected in human populations, initially in Great Britain (known variant Alpha, B.1.1.7 lineage), South Africa (Beta, B.1.351 lineage) and India (Delta, B.1.617.2 lineage) in December 2020, Brazil (Gamma, P.1 lineage) in January 2021, and South Africa in November 2021 (omicron, B.1.1.529 lineage). They subsequently spread all over the world [2,3]. Only vaccines are efficient against COVID-19 and prevent both severe cases and deaths due to SARS-CoV-2 infection [4,5]. Currently, very few treatments are recommended against SARS-CoV-2. Therefore, the identification of effective drugs among FDA-approved drugs could be a rapid, efficient and low-cost strategy against SARS-CoV-2. Several repurposing drugs have already been evaluated in vitro, including antimalarial drugs [6,7,8,9], antibiotics [10,11], antivirals [8,9,12,13], anti-leprosy drugs [14], antipsychotics [15], antihistaminics [16,17], immunosuppressive agents [18,19] and other pharmacological agents [20,21]. Other repurposing drugs exhibited anti-SARS-CoV-2 activity in combination by promoting the absorption of partner-like N-acetyl cysteine [22]. 

One of those drugs is ivermectin. It is an antihelminthic agent that previously showed in vitro effects on RNA and DNA viruses such as Zika virus, dengue virus, West Nile virus, Chikungunya virus and equine herpesvirus type I [23]. Ivermectin, used alone or in combination with remdesivir, reduced the viral load in mice infected with murine hepatis virus (MHV), a coronavirus that infects mice and shares a sequence identity with SARS-CoV-2 [24,25]. The in vitro evaluation of ivermectin was described in only one paper using only one SARS-CoV-2 isolate (Australia/VI01/2020 isolate). Ivermectin showed antiviral in vitro activity against SARS-CoV-2, with a median inhibitory concentration (IC_50_) of around 2.0 µM [26].

However, infectivity, clinical severity, re-infection, virulence, transmissibility, vaccine responses and escape, and epidemiological aspects have differed according to the SARS-CoV-2 variants [27,28,29,30,31,32,33,34,35,36,37]. The aim of this study was to evaluate the in vitro antiviral activity of ivermectin compared to chloroquine and remdesivir against 30 strains of SARS-CoV-2 isolated from patients infected with different variants of concern (VOCs) (alpha, beta, delta, gamma, omicron) and variants of interest (VOIs) (Marseille-1, Marseille-4…), and to analyse their antiviral susceptibility and to determine whether the in vitro efficacy of ivermectin differs according to isolates and variants.

## 2. Results

**Figure 1:**
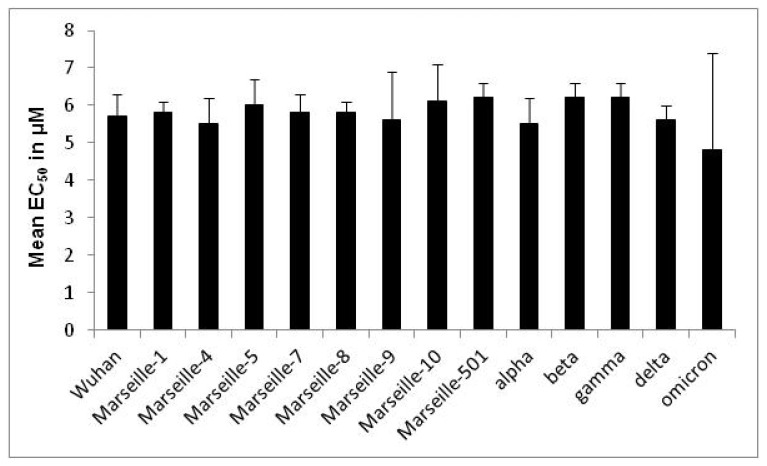
EC_50_ means of ivermectin according to the 14 clinically isolated variants of SARS-CoV-2 (error bar represents the standard deviation of 5 to 11 independent experiments).

The EC_50_ means of ivermectin for the 30 clinically isolated SARS-CoV-2 strains ranged from 1.3 ± 0.5 to 6.7 ± 0.4 µM (Table 1). There was no significant difference regarding ivermectin activity within a variant (related to Wuhan, Marseille-1, Marseille-4, alpha or delta) regardless of the strain tested (*p* from 0.09 to 0.29, Kruskal–Wallis rank sum test) (Table 1). Only the omicron variant showed a significant different susceptibility between the different strains (EC_50_ means ranged from 1.3 ± 0.5 to 6.7 ± 0.4 µM, *p* = 0.003). There was a significant difference between the variants analysed (*p* = 0.0002, Kruskal–Wallis rank sum test) (Figure 1).

The EC_50_ means of chloroquine, used for comparison, for the 30 clinically isolated SARS-CoV-2 strains ranged from 4.3 ± 2.5 to 33.7 ± 9.0 µM (Table 2).

**Figure 2:**
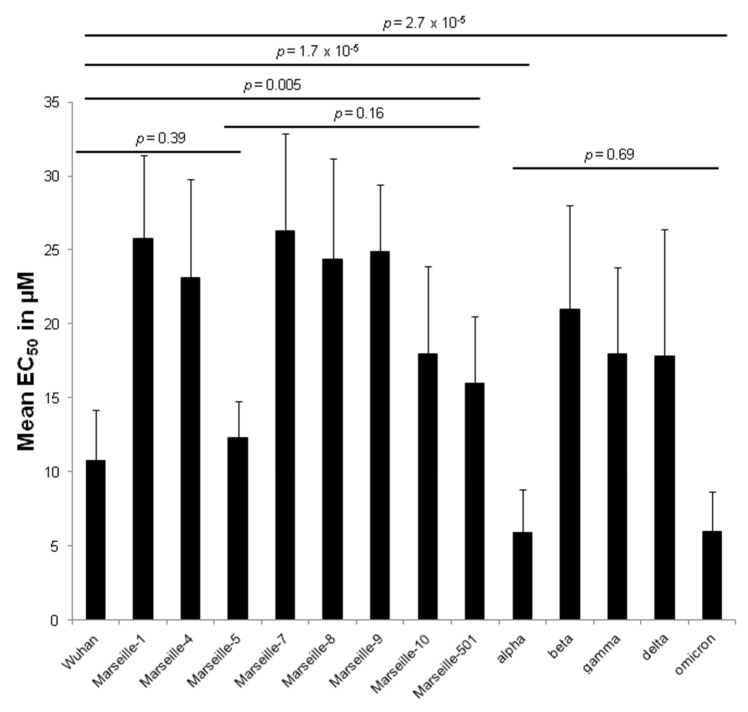
EC_50_ means of chloroquine according to the 14 clinically isolated variants of SARS-CoV-2 (error bar represents the standard deviation of 5 to 11 independent experiments).

There was no significant difference in chloroquine activity within a variant (related to Wuhan, Marseille-1, Marseille-4, alpha, delta or omicron) regardless of the strains tested (*p* from 0.06 to 0.82, Kruskal–Wallis rank sum test) (Table 2). However, there was a significant difference between the different variants analysed (*p* = 1.4 × 10^−22^, Kruskal–Wallis rank sum test). The alpha and omicron variants were the most susceptible (*p* = 1.7 × 10^−5^ and 2.7 × 10^−5^, respectively, compared to the original Wuhan variant) (Figure 2). There was no significant difference in chloroquine susceptibility between the omicron and alpha variants (*p* = 0.69).

The EC_50_ means of remdesivir, used for comparison, for the 30 clinically isolated SARS-CoV-2 strains ranged from 0.4 ± 0.3 to 25.9 ± 7.4 µM (Table 3).

**Figure 3:**
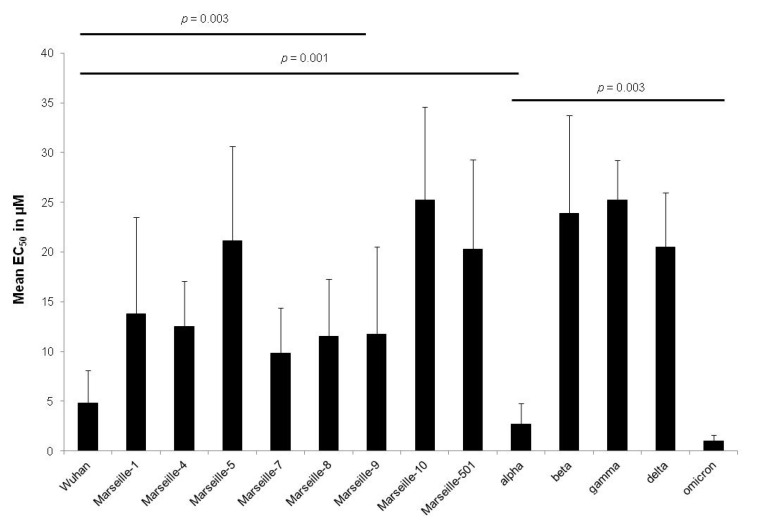
EC_50_ means of remdesivir according to the 14 clinically isolated variants of SARS-CoV-2 (error bar represents the standard deviation of 5 to 11 independent experiments).

There was no significant difference in remdesivir activity within a variant (related to Wuhan, Marseille-1, Marseille-4, alpha or delta) regardless of the strains tested (*p* from 0.08 to 0.94, Kruskal–Wallis rank sum test) (Table 3). Only the omicron variant showed significant susceptibilities between the different strains (EC_50_ means ranged from 0.4 ± 0.3 to 1.3 ± 0.1 µM, *p* = 0.006). There was a significant difference between the different variants analysed (*p* = 2.0 × 10^−21^, Kruskal–Wallis rank sum test). The omicron variant was the most susceptible to remdesivir (*p* = 0.003, omicron variant versus alpha variant) followed by the alpha variant (*p* = 0.01, alpha variant versus variant related to Wuhan) and by the variant related to Wuhan (*p* = 0.03, alpha variant versus Marseille-9 variant) (Figure 3).

## 3. Discussion

Although the 14 SARS-CoV-2 variants showed a significant variation of in vitro susceptibility to ivermectin (from 4.8 to 6.2 µM, *p* = 0.0002), these were relatively homogeneous (from 5.1 ± 0.5 to 6.7 ± 0.4 µM) if the susceptibility of the IHU-MI-5227 omicron strain is removed. Indeed, this strain presented a higher susceptibility to ivermectin than the other 29 strains (1.3 ± 0.5 µM). These results were consistent with the previous data by Caly et al. (IC_50_ = 2.8 µM) [26]. Ivermectin was potent in vitro against SARS-CoV-2, regardless of the strains and the variants. Ivermectin (No. EC_50_ = 219, mean EC_50_ = 5.7 ± 1.0 µM) was more potent in vitro overall than chloroquine (No. EC_50_ = 214, mean EC_50_ = 16.1 ± 9.0 µM) (*p* = 1.3 × 10^−34^, Welch *t*-test) and remdesivir (No. EC_50_ = 201, mean EC_50_ = 11.9 ± 10.0 µM) (*p* = 1.6 × 10^−13^, Welch *t*-test).

Several modes of action were suggested for ivermectin [38]. Host proteins, such as STAT transcription factors, interact with the importin heterodimer complex (IMPα/β1) by binding the IMPα in cytoplasm and are transported into the nucleus using the nuclear pore complex (NPC) located in the nuclear envelope [39]. Many viruses interact with IMPα/β1 to access into the nucleus through the NPC [40]. Ivermectin has been found to reduce West Nile, dengue, HIV-1 and influenza A viral replication by inhibiting nuclear import via IMPα/β1 [41,42,43,44]. It was suggested that ivermectin also decreases SARS-CoV-2 replication by inhibiting IMPα/β1-mediated nuclear transport [26]. In silico molecular docking reports interaction between ivermectin and IPMα [45,46,47]. Another hypothesis is the inhibition of the viral RNA-dependent RNA polymerase (RdRp, replicase) which is essential for viral genome replication. A strong interaction between ivermectin and SARS-CoV-2 RdRp has been demonstrated by the in silico approach [48,49,50,51]. In some studies, ivermectin showed higher binding affinity to the predicted active RdRp than remdesivir [48,49,50], which is known to inhibit viral replication and RdRp [52,53]. Another replicase, named 3 chymotrypsin-like protease (3CLpro) or main protease (Mpro) is crucial in SARS-CoV-2 replication, leading to the formation of non-structural proteins (NSPs) [54]. Ivermectin has been found to have a strong interaction with 3C-like protease [46,47,49,51,55,56]. Ivermectin could also inhibit SARS-CoV-2 cell entry by linking itself to the SARS-CoV-2 viral spike glycoprotein receptor-binding domain (RBD) and the angiotensin-converting enzyme-2 (ACE2) transmembrane receptor protein [45,46,57,58]. Moreover, SARS-CoV-2 requires the transmembrane protease serine 2 (TMPPRSS2) in order to activate the spike protein. This protein can also be a potential target for ivermectin [48,51].

Unlike ivermectin, which showed relatively homogeneous in vitro activity against SARS-CoV-2 regardless of the strain or variant, variant susceptibility to chloroquine was heterogeneous. Omicron and alpha variants were the most susceptible to chloroquine. The strains related to Wuhan were less susceptible than strains belonging to the omicron and alpha variants but were more susceptible than strains belonging to the beta, gamma and delta variants. Chloroquine can inhibit in silico viral entry into the host cell by interacting with sialic acids linked to gangliosides on host cellular surface and ACE receptor [59,60,61,62,63]. In the presence of chloroquine, the viral spike protein is no longer able to link gangliosides [59]. Chloroquine also interacts with the TMPRSS2 protein [51,64,65]. The replicase 3CLpro may also be a potential target for chloroquine [51,55,66,67].

The emergence of mutations in the spike glycoprotein of SARS-CoV-2 might impact drug efficacy, and more particularly chloroquine efficacy. As compared to the spike protein relative to the Wuhan sequences, the different variants include few non-synonymous mutations except the omicron variant which contains 30 mutations. The different mutations for each variant are reported in Supplementary Table S1. The significant polymorphism of the omicron spike protein would suggest changes in protein structure and a decrease in chloroquine in vitro activity. Conversely, the omicron variant is one of the two most susceptible variants. Recently, it has been shown that omicron enters cells mainly by TMPRSS2-independent fusion following endocytosis after processing by cathepsin B or L, while the other variants enter by fusion following proteolytic processing by TMPPRSS2 [68]. Chloroquine is a weak base compound, referred to as lysosomotropic drug, which accumulates in endosomes and lysosomes, and increased lysosomal pH leading to a decrease in lysosomal protease activities and, finally, prevents viral entry into host cells [69,70]. Chloroquine also inhibits viral replication due to the lack of enzyme functional activities at a high pH [71]. The elevated pH in endosomes by chloroquine could explain the in vitro activity against the omicron variant.

In vitro susceptibility to remdesivir also varied. The three omicron, alpha and Wuhan-related variants were the most susceptible variants. In vitro remdesivir shows a broad spectrum of antiviral activity against RNA viruses by targeting replicase such as RdRp [52,72]. Remdesivir can also inhibit the SARS-CoV-2 RdRp [51,53,73,74,75]. Remdesivir can also dock the 3CLpro replicase [49,51,76,77]. These results are consistent with in vitro data demonstrating that it inhibits SARS-CoV-2 viral replication only at the post-entry stage in Vero E6 cells and not at the entry stage [21].

However, these results must be interpreted with caution regarding the potential use of ivermectin in SARS-CoV-2-infected patients: it is difficult to translate in vitro study results into actual clinical treatment in patients. First, it is crucial to determine whether the concentrations required are consistent with concentrations observed in humans. Ninety-three percent of ivermectin is bound to plasma proteins and there is no data on penetration and concentration of ivermectin into human lungs [78]. Modelling the FDA-approved dose of 200 µg/kg or single oral dose of 120 mg leads to insufficient concentrations in plasma or lung tissue to achieve around 5 µM [79,80,81]. After a single dose of ivermectin 200 µg/kg, lung concentrations are predicted to be around a quarter of an IC_50_ of around 2.0 µM [82]. Another model, considering host viral kinetics of SARS-CoV-2, pharmacodynamic effects and the pharmacokinetic profile of ivermectin, shows that ivermectin at 600 µg/kg three times a day in a patient weighing 70 kg has similar effects to the maximal oral dose of 120 mg and significantly reduced SARS-CoV-2 viral load [82]. Moreover, Arshad et al. predicted ivermectin accumulation in lung tissue over 20 times higher than EC_50_ [83].

Ivermectin antiviral activity can be improved by combinations with antiviral agents with differing modes of action and new pharmaceutical formulations that can more efficiently deliver ivermectin at high concentrations in the lung tissue. The in vitro combination of ivermectin (2 µM) and remdesivir (6 µM) shows highly synergistic effects against the murine hepatitis virus (MHV), which belongs to the betacoronavirus genus like the SARS-CoV-2 [25]. Ivermectin exerts higher in vitro inhibition of importin α nuclear accumulation in combination with atorvastatin than when used alone [84]. A randomised, blind trial in patients with mild-to-moderate COVID-19 symptoms showed that patients treated with ivermectin 12 mg and doxycycline 100 mg, twice a day for five days, recovered earlier than those receiving standard care alone (paracetamol, antihistaminics, vitamins, low molecular weight heparin and oxygen therapy if necessary), and were more significantly asymptomatic after 12 days and were less likely to be diagnosed with SARS-CoV-2 after 14 days [85]. However, the viral load was not estimated and the efficacy of ivermectin or doxycycline used alone was not evaluated. Another pilot clinical trial in patients with mild-to-moderate COVID-19 symptoms showed that patients who received a single dose of ivermectin 200 µg/kg at the day of admission in combination with hydroxychloroquine (400 mg twice a day for the first day and 200 mg twice a day for five days) associated with azithromycin (500 mg the first day and 250 mg for five days) were cured faster and had a shorter stay in hospital in comparison with patients who received only hydroxychloroquine in combination with azithromycin [86]. However, these results must be interpreted with caution given the small sample size and because this study was not randomised.

The administration of ivermectin by inhalation could be a way to achieve its accumulation into the lungs. The administration of nebulised ivermectin at 116.5 mg/kg in rats led to plasma concentrations of 186.7 ng/mL (0.21 µM) 24h after administration and 524.3 ng/g in lung tissue 168 h after administration [87]. In piglets, after the administration of one dose of 2 mg of ivermectin by nasal spray, a significant positive correlation was reported between the ivermectin concentrations in nasopharyngeal and lung tissues [88]. Ivermectin concentrations in nasopharyngeal tissue may be higher with an intranasal dose of ivermectin 2 mg twice a day than a single oral dose of 0.2 mg/kg (12 mg) for a person weighing 60 kg.

The clinical efficacy of ivermectin in COVID-19 treatments remains controversial. In one retrospective study, a single standard dose of 200 µg/kg of ivermectin did not significantly reduce the duration of the SARS-CoV-2 detection and did not improve clinical outcomes in severe COVID-19 patients [89]. However, no difference was found in baseline characteristics, clinical presentation, use of associated treatment (such as hydroxychloroquine, azithromycin, lopinavir, ritonavir, remdesivir, tocilizumab or beta-interferon) and outcomes between patients treated with and without ivermectin. Moreover, a daily dose of 14 mg of ivermectin for four days did not significantly reduce the need for admission to an intensive care unit, the use of invasive ventilation or the occurrence of death in patients hospitalised with severe COVID-19, in comparison with treatment with hydroxychloroquine (400 mg daily for five days) [90]. Unfortunately, the efficacy of ivermectin was not compared against a placebo. There was no difference in RT-PCR negativity on day 6 between patients who received a daily dose of 12 mg of ivermectin for two days compared to patients who did not receive ivermectin treatment [91]. However, a daily dose of 12 mg of ivermectin for two days significantly prevented mortality (100% of patients were successfully discharged compared to 93%). In another randomised, double-blind, placebo-controlled trial (IVERCOR-COVID-19) using the same regimen, ivermectin administration did not improve RT-PCR on days 3 and 7 and did not prevent hospitalisation [92]. A five-day course of 300 µg/kg of ivermectin per day (compared to a placebo) did not significantly improve the time of recovery from symptoms (10 days compared to 12 days) [93]. In a pilot, double blind, randomised controlled trial in hospitalised patients with mild-to-moderate manifestations of COVID-19, a single oral administration of ivermectin of either 12 or 24 mg did not significantly reduce the viral load on day 5 or negate the presence of SARS-CoV-2 in comparison with a placebo, although RT-PCR negativity was higher but not significant in the group of patients who received 24 mg (47.5%) than those who received a placebo (31.1%) [94]. Oral ivermectin used at 400 µg/kg body weight daily for five days in addition to standard clinical care did not prevent progression to severe disease among high-risk patients with mild to moderate COVID-19 in comparison with patients who received only standard care (21.6% versus 17.3%) [95]. The use of a single dose of 12 mg of ivermectin in combination with azithromycin, montelukast (a cysteinyl leukotriene receptor antagonist) and acetylsalicylic acid improved recovery and prevented the risk of hospitalisation and death in COVID-19 out-patients compared to the placebo group [96]. However, the patients who received ivermectin had a significantly lower prevalence of comorbidities and were younger than the comparison group. Moreover, it is difficult to evaluate the role of ivermectin in the efficacy of the combination. In another randomised, double-blind, placebo-controlled pilot trial in patients with non-severe manifestations of COVID-19 and no risk factors for complicated disease, individuals treated with a single dose of 400 µg/kg of ivermectin had lower but not significantly lower viral loads on day 4 and day 7 post-treatment [97]. Patients treated with ivermectin recovered earlier from hyposmia, anosmia and a cough. Patients who received two doses of 200 µg/kg of ivermectin in addition to standard clinical care stayed in intensive care for a significantly shorter time (three days versus 18 days) and required a shorter duration of mechanical ventilation (three days versus 18 days) than the control group who received only standard clinical care [98]. A reduction of 11.2% in the risk of death was reported in hospitalised patients treated with a single oral dose of 200 µg/kg of ivermectin in addition to standard clinical care, compared with patients only treated with standard clinical care [99]. Most of the clinical studies previously cited were reanalysed to assess their risk of bias including randomisation, blinding, attrition or estimation of effects and were classified as studies with a low risk of bias [100,101]. Most of the previous publications or systematic reviews concluding that there were significant benefits of the use of ivermectin were based on potentially biased results due to methodological limitations [101,102]. Further stringent research is needed.

## 4. Materials and Methods

### 4.1. Virus Collection, Cells and Drugs

Ivermectin and chloroquine diphosphate were bought from Sigma Aldrich (St Quentin Fallavier, France) and remdesivir from Apollo Scientific (Manchester, UK). Stock solutions of ivermectin and remdesivir were prepared in DMSO/water 10% and chloroquine in water. All the stock solutions were then diluted in Minimum Essential Media (MEM, Gibco, ThermoFisher, Waltham, MA, USA) in order to have seven final concentrations ranging from 0.1 µM to 100 µM. Final concentrations of DMSO in the assay were under 0.2% and had no influence in viral replication into Vero E6 cells.

Thirty clinically isolated SARS-CoV-2 strains were used: five strains closely related to the initial Wuhan isolate (IHU-MI-003, IHU-MI-006, IHU-MI-717, IHU-MI-845 and IHU-MI-847) (B lineage) were collected from hospitalised patients during the first COVID-19 outbreak in March–May 2020 in Marseille [103], four strains (IHU-MI-2122, IHU-MI-2123, IHU-MI-2177 and IHU-MI-2178) belonging to the Marseille-1 variant (B.1.416 lineage) originating from Algeria were collected from patients in July–August 2020 [104], three strains (IHU-MI-2096, IHU-MI-2129 and IHU-MI-2179) belonging to the Marseille-4 variant (B.1.160 lineage) originating from a mink farm in Eure et Loire (France) were collected from patients in July 2020 [105], one strain (IHU-MI-2137) belonging to the Marseille-5 variant ((B.1.367 lineage) [105], one strain (IHU-MI-2519) belonging to the Marseille-7 variant [105], one strain (IHU-MI-2555) belonging to the Marseille-8 variant [105], one strain (IHU-MI-2615) belonging to the Marseille-9 variant (B.1.1.241 lineage) [105], one strain (IHU-MI-2403) belonging to the Marseille-10 variant [105], one strain (IHU-MI-3217) belonging to the Marseille-501 variant (A.27 lineage) including the N501Y mutation in the spike protein was collected from a patient from Comoros in January 2021 [106], four strains (IHU-MI-3076, IHU-MI-3100, IHU-MI-3127 and IHU-MI-3128) belonging to the alpha variant (B.1.1.7 lineage) originating from the UK [103], one strain (IHU-MI-3147) belonging to the beta variant (B.1.351 lineage) originating from South Africa [103], one strain (IHU-MI-3191) belonging to the gamma variant (P.1 lineage) originating from Brazil [103], three strains (IHU-MI-3396, IHU-MI-3630 and IHU-MI-4654) belonging to the delta variant (B.1.617 lineage) originating from India [107] and three strains (IHU-MI-5227, IHU-MI-5245 and IHU-MI-5253) belonging to the omicron variant (B.1.1.529 lineage) originating from South Africa.

The strains were maintained in production in Vero E6 cells (American type culture collection ATCC^®^ CRL-1586™) in MEM with 4% foetal bovine serum and 1% glutamine (complete medium). Vero E6 cells are one of the most widely used cells for the culture of SARS-CoV-2 due to the presence of the high expression of angiotensin converting enzyme 2 (ACE2) receptors, essential for SARS-CoV-2 cell entry [108,109]. Vero E6 cells were found to be relevant for antiviral drug screening models [17,109].

### 4.2. Antiviral Activity Assay

Briefly, 96-well plates were prepared with 5 × 10^5^ cells/mL of Vero E6 (200 µL per well), as previously described [10]. The different concentrations of ivermectin, chloroquine or remdesivir were added 4 h before infection. The replication of the different strains in Vero E6 cells at an MOI of 0.01 was estimated 48 h after infection by RT-PCR using the Superscript III platinum one step with Rox kit (Invitrogene, Villebon sur Yvette, France) after RNA extraction using the QIAamp 96 Virus QIAcube HT Kit (QIAGEN, Hilden, Germany) on the QIAcube HT System (QIAGEN, Hilden, Germany). The primers used were previously described [110]. The percentage of inhibition of SARS-CoV-2 replication was estimated for each drug concentration as follows: (mean CT_drug concentration_ − mean CT_control 0%_)/(mean CT_control 100%_ − mean CT_control 0%_) × 100. The CT_control 0%_ (0% of inhibition) corresponds to the mean of 12 CT of SARS-CoV-2 replication in the absence of drug 48 h after infection. The CT_control 100%_ (100% of inhibition) corresponds to the mean of 12 CT of SARS-CoV-2 after 48 h of Vero E6 cells infection in the presence of high concentrations of drug. This CT_control 0%_ is similar to the CT of virus inoculum used to infect Vero E6 cells at 0 h.

EC_50_ (median effective concentration) values were estimated through nonlinear regression using the R software (ICEstimator version 1.2). EC_50_ values resulted in the mean of 5 to 11 independent experiments.

## 5. Conclusions

The in vitro evaluation of ivermectin has been described in only one paper using only one SARS-CoV-2 isolate (Australia/VI01/2020 isolate) [26]. In the present work, we proposed to compare the in vitro activity of ivermectin first between various isolates belonging to the same variant and then between various variants (and more particularly variants related to Wuhan, alpha, beta, gamma, delta and omicron). Unlike chloroquine or remdesivir, ivermectin showed a relatively homogeneous in vitro activity against SARS-CoV-2 (4.8 to 6.2 µM) regardless of the strain or variant of concern (Wuhan, alpha, beta, gamma, delta or omicron). These results must be interpreted with caution regarding the potential use of ivermectin in SARS-CoV-2-infected patients: it is difficult to translate in vitro study results into actual clinical treatment in patients. The expected ivermectin concentration levels in human lungs after standard doses remain controversial, as well as its efficacy in patients with COVID-19. Ivermectin antiviral activity can be improved by combinations with antiviral agents with differing modes of action and new pharmaceutical formulations that can more efficiently deliver ivermectin at high concentrations into the lung tissue (through inhalation, for instance). Further stringent research, particularly clinical trials, is needed to investigate ivermectin as COVID-19 treatment.

## Figures and Tables

**Table 1:** In vitro susceptibility of different clinically isolated SARS-CoV-2 variants of concern or interest to ivermectin.

Variant	Origin	Strain Name	EC_50_ in µM (Mean ± SD ^a^)	Variant EC_50_ in µM (Mean ± SD ^a^)	*p*-Value
	Wuhan	IHU-MI-003	5.8 ± 0.5	5.7 ± 0.6	0.09
		IHU-MI-006	5.9 ± 0.7		
		IHU-MI-717	6.1 ± 0.4		
		IHU-MI-845	5.1 ± 0.5		
		IHU-MI-847	5.4 ± 0.5		
Marseille-1	Algeria	IHU-MI-2122	5.3 ± 1.7	5.8 ± 0.3	0.10
		IHU-MI-2123	5.9 ± 0.4		
		IHU-MI-2177	5.5 ± 0.1		
		IHU-MI-2178	5.9 ± 0.4		
Marseille-4	France	IHU-MI-2096	5.7 ± 0.4	5.5 ± 0.7	0.29
		IHU-MI-2129	5.5 ± 0.2		
		IHU-MI-2179	5.1 ± 1.1		
Marseille-5		IHU-MI-2137	6.0 ± 0.7	6.0 ± 0.7	
Marseille-7		IHU-MI-2519	5.8 ± 0.5	5.8 ± 0.5	
Marseille-8		IHU-MI-2555	5.8 ± 0.3	5.8 ± 0.3	
Marseille-9		IHU-MI-2615	5.6 ± 1.3	5.6 ± 1.3	
Marseille-10		IHU-MI-2403	6.1 ± 1.0	6.1 ± 1.0	
Marseille-501	Comoros	IHU-MI-3217	6.2 ± 0.4	6.2 ± 0.4	
alpha	UK	IHU-MI-3076	5.8 ± 0.8	5.5 ± 0.7	0.10
		IHU-MI-3100	5.8 ± 0.7		
		IHU-MI-3127	5.2 ± 0.4		
		IHU-MI-3128	5.1 ± 0.6		
beta	South Africa	IHU-MI-3147	6.2 ± 0.4	6.2 ± 0.4	
gamma	Brazil	IHU-MI-3191	6.2 ± 0.4	6.2 ± 0.4	
delta	India	IHU-MI-3396	5.6 ± 0.2	5.6 ± 0.4	0.70
		IHU-MI-3630	5.7 ± 0.6		
		IHU-MI-4654	5.6 ± 0.4		
omicron	South Africa	IHU-MI-5227	1.3 ± 0.5	4.8 ± 2.6	0.003
		IHU-MI-5245	6.7 ± 0.4		
		IHU-MI-5253	6.3 ± 0.5		

^a^ SD = standard deviation.

**Table 2:** In vitro susceptibility of different clinically isolated SARS-CoV-2 variants of concern and interest to chloroquine.

Variant	Origin	Strain Name	EC_50_ in µM (Mean ± SD ^a^)	Variant EC_50_ in µM (Mean ± SD ^a^)	*p*-Value
	Wuhan	IHU-MI-003	8.9 ± 4.4	10.8 ± 3.4	0.21
		IHU-MI-006	11.4 ± 2.4		
		IHU-MI-717	12.4 ± 4.0		
		IHU-MI-845	7.7 ± 1.5		
		IHU-MI-847	11.7 ± 1.9		
Marseille-1	Algeria	IHU-MI-2122	25.5 ± 5.2	25.8 ± 5.6	0.59
		IHU-MI-2123	24.6 ± 6.1		
		IHU-MI-2177	29.3 ± 5.2		
		IHU-MI-2178	23.8 ± 7.1		
Marseille-4	France	IHU-MI-2096	21.9 ± 5.7	23.1 ± 6.7	0.82
		IHU-MI-2129	24.1 ± 8.3		
		IHU-MI-2179	24.6 ± 4.5		
Marseille-5		IHU-MI-2137	12.3 ± 2.5	12.3 ± 2.5	
Marseille-7		IHU-MI-2519	26.3 ± 6.6	26.3 ± 6.6	
Marseille-8		IHU-MI-2555	24.4 ± 6.8	24.4 ± 6.8	
Marseille-9		IHU-MI-2615	24.9 ± 4.5	24.9 ± 4.5	
Marseille-10		IHU-MI-2403	18.0 ± 5.9	18.0 ± 5.9	
Marseille-501	Comoros	IHU-MI-3217	16.0 ± 4.5	16.0 ± 4.5	
alpha	UK	IHU-MI-3076	8.4 ± 2.8	5.9 ± 2.9	0.06
		IHU-MI-3100	6.5 ± 1.8		
		IHU-MI-3127	4.4 ± 2.7		
		IHU-MI-3128	4.3 ± 2.5		
beta	South Africa	IHU-MI-3147	21.0 ± 7.0	21.0 ± 7.0	
gamma	Brazil	IHU-MI-3191	18.0 ± 5.8	18.0 ± 5.8	
delta	India	IHU-MI-3396	22.0 ± 11.3	17.8 ± 8.6	0.48
		IHU-MI-3630	14.3 ± 2.8		
		IHU-MI-4654	15.0 ± 5.6		
omicron	South Africa	IHU-MI-5227	5.2 ± 2.0	6.0 ± 2.7	0.06
		IHU-MI-5245	8.2 ± 2.1		
		IHU-MI-5253	4.5 ± 2.4		

^a^ SD = standard deviation.

**Table 3:** In vitro susceptibility of different clinically isolated SARS-CoV-2 variants of concern or interest to remdesivir.

Variant	Origin	Strain Name	EC_50_ in µM (Mean ± SD ^a^)	Variant EC_50_ in µM (Mean ± SD ^a^)	*p*-Value
	Wuhan	IHU-MI-003	2.7 ± 1.6	4.8 ± 3.3	0.61
		IHU-MI-006	4.3 ± 1.6		
		IHU-MI-717	7.3 ± 4.9		
		IHU-MI-845	4.7 ± 2.7		
		IHU-MI-847	3.7 ± 2.8		
Marseille-1	Algeria	IHU-MI-2122	12.9 ± 7.6	13.8 ± 9.7	0.08
		IHU-MI-2123	5.1± 1.2		
		IHU-MI-2177	21.8 ± 8.0		
		IHU-MI-2178	16.4 ± 8.6		
Marseille-4	France	IHU-MI-2096	12.2 ± 2.7	12.5 ± 4.6	0.94
		IHU-MI-2129	12.5 ± 7.3		
		IHU-MI-2179	13.0 ± 7.4		
Marseille-5		IHU-MI-2137	21.1 ± 9.5	21.1 ± 9.5	
Marseille-7		IHU-MI-2519	9.8 ± 4.6	9.8 ± 4.6	
Marseille-8		IHU-MI-2555	11.5 ± 5.8	11.5 ± 5.8	
Marseille-9		IHU-MI-2615	11.7 ± 8.8	11.7 ± 8.8	
Marseille-10		IHU-MI-2403	25.2 ± 9.4	25.2 ± 9.4	
Marseille-501	Comoros	IHU-MI-3217	20.3 ± 9.0	20.3 ± 9.0	
alpha	UK	IHU-MI-3076	3.5 ± 2.2	2.7 ± 2.1	0.77
		IHU-MI-3100	2.0 ± 1.0		
		IHU-MI-3127	3.4 ± 2.8		
		IHU-MI-3128	2.6 ± 2.0		
beta	South Africa	IHU-MI-3147	23.9 ± 9.8	23.9 ± 9.8	
gamma	Brazil	IHU-MI-3191	25.2 ± 4.0	25.2 ± 4.0	
delta	India	IHU-MI-3396	21.4 ± 5.5	20.5 ± 5.5	0.71
		IHU-MI-3630	21.9 ± 2.7		
		IHU-MI-4654	17.6 ± 6.9		
omicron	South Africa	IHU-MI-5227	0.4 ± 0.3	1.0 ± 0.6	0.006
		IHU-MI-5245	1.2 ± 0.4		
		IHU-MI-5253	1.3 ± 0.1		

^a^ SD = standard deviation.

## Data Availability

The analysed data presented in this study are available on the main text and the raw data are available on request from the corresponding author. The raw data are not publicly available due to due to archiving on a military server.

## Supplementary Materials

The following supporting information can be downloaded at: https://www.mdpi.com/article/10.3390/ph15040445/s1, Table S1: List of nucleotide and amino acid changes associated with the different SARS-CoV-2 variants.
