# Supplementary material for: Antiviral Activity of Repurposing Ivermectin against a Panel of 30 Clinical SARS-CoV-2 Strains Belonging to 14 Variants
Source: Pharmaceuticals (Basel). 2022 Apr 2;15(4):445. doi: 10.3390/ph15040445 (PMC9024598; doi:10.3390/ph15040445)
Supplement: Supplementary file 1 [file pharmaceuticals-15-00445-s001.zip › pharmaceuticals-1604607-supplementary.pdf]

Table S1: List of nucleotide and amino acid changes associated with the different SARS-CoV-2 variants

Variant	Sequence name	clade	substitutions	deletions	Amino acid substitutions	Amino acid deletions
Wuhan	Wuhan	19A				
Marseille-1	20A/20268G.MRS-1	20A	C241T,G1181T,C1625T,C3037T,C14408T,A20268G,G22894A,A23403G,C25886T,G28198T,C28833T,G28851T		N:S187L,N:S193I,ORF1a:V306F,ORF1a:L454F,ORF1b:P314L,ORF3a:S165F,ORF8:C102F,S:D614G	
Marseille-4	20A/25563T.MRS-4	20A	C241T,C3037T,C4543T,G5629T,G9526T,C11497T,G13993T,C14408T,G15766T,A16889G,G17019T,C18877T,G22992A,A23403G,G25563T,C25710T,C26735T,T26876C,G28975C,G29399A		N:M234I,N:A376T,ORF1a:M3087I,ORF1b:A176S,ORF1b:P314L,ORF1b:V767L,ORF1b:K1141R,ORF1b:E1184D,ORF3a:Q57H,S:S477N,S:D614G	
Marseille-5	20C.MRS-5	20C	C241T,C1059T,C3037T,C3099T,G4960T,C4965T,C6070T,C7303T,C7564T,C9246T,C10279T,C10301A,C10525T,C10582T,G10688T,G11851T,C14230A,C14408T,G21800T,A23403G,G25563T,G27632T,C27804T,C28830A,G29402T,G29779T		N:S186Y,N:D377Y,ORF1a:T265I,ORF1a:T945I,ORF1a:T1567I,ORF1a:A2994V,ORF1a:Q3346K,ORF1a:V3475F,ORF1a:M3862I,ORF1b:P255T,ORF1b:P314L,ORF3a:Q57H,ORF7a:R80I,S:D80Y,S:D614G	
Marseille-7	20A/20268G.MRS-7	20A	C241T,C2706T,C3037T,C14408T,A20268G,A23403G,C25731T,G27463C,C28833T		N:S187L,ORF1a:T814I,ORF1b:P314L,ORF7a:V24L,S:D614G	
Marseille-8	20B.MRS-8	20B	C241T,C3037T,C5055T,G11851T,A12755G,C14408T,A23403G,G24812T,C26895T,G28881A,G28882A,G28883C		M:H125Y,N:R203K,N:G204R,ORF1a:T1597I,ORF1a:M3862I,ORF1a:T4164A,ORF1b:P314L,S:D614G,S:D1084Y	
Marseille-9	20B.MRS-9	20B	C241T,C3037T,A11782G,C14408T,T21570G,C21575T,A23403G,T25473C,C28253T,G28881A,G28882A,G28883C		N:R203K,N:G204R,ORF1b:P314L,S:V3G,S:L5F,S:D614G	
Marseille-10	20A/15324T.MRS-10	20A	C241T,C3037T,C3602T,C6941T,C14408T,C15324T,C21855T,A23403G,A25505G,G25906C,G25996T,C28651T,C28869T		N:P199L,ORF1a:H1113Y,ORF1b:P314L,ORF3a:Q38R,ORF3a:G172R,ORF3a:V202L,S:S98F,S:D614G	
Marseille-501	19B.MRS-501Y	19B	A361G,C1122T,C2509T,C8782T,A9204G,A11217G,C16466T,A18366G,A20262G,T22917G,A23063T,C23520T,C23525T,G23948T,G25218T,T25541C,C27247T,T28144C,A28273T,G28878A,G29742A	26161-26168,27388,28248-28253	N:S202N,ORF1a:P286L,ORF1a:D2980G,ORF1a:N3651S,ORF1b:P1000L,ORF3a:V50A,ORF8:L84S,S:L452R,S:N501Y,S:A653V,S:H655Y,S:D796Y,S:G1219V	ORF3a:N257-,ORF3a:P258-,ORF8:D119-,ORF8:F120-
Alpha	20I	20I (Alpha, V1)	C241T,C913T,C3037T,C3267T,C5388A,C5986T,T6954C,C14408T,C14676T,C15279T,T16176C,A23063T,C23271A,A23403G,C23604A,C23709T,T24506G,G24914C,C27972T,G28048T,A28111G,G28280C,A28281T,T28282A,G28881A,G28882A,G28883C,C28977T	11288-11296,21765-21770,21992-21994,28271	N:D3L,N:R203K,N:G204R,N:S235F,ORF1a:T1001I,ORF1a:A1708D,ORF1a:I2230T,ORF1b:P314L,ORF8:Q27*,ORF8:R52I,ORF8:Y73C,S:N501Y,S:A570D,S:D614G,S:P681H,S:T716I,S:S982A,S:D1118H	ORF1a:S3675-,ORF1a:G3676-,ORF1a:F3677-,S:H69-,S:V70-,S:Y144-
Beta	20H	20H (Beta, V2)	G174T,C241T,C1059T,C3037T,G5230T,A10323G,C14408T,A21801C,A22206G,G22813T,G23012A,A23063T,A23403G,C23664T,G25563T,C25904T,C26456T,C28253T,C28887T	11288-11296,22283-22291	E:P71L,N:T205I,ORF1a:T265I,ORF1a:K1655N,ORF1a:K3353R,ORF1b:P314L,ORF3a:Q57H,ORF3a:S171L,S:D80A,S:D215G,S:K417N,S:E484K,S:N501Y,S:D614G,S:A701V	ORF1a:S3675-,ORF1a:G3676-,ORF1a:F3677-,S:L241-,S:L242-,S:A243-
Gamma	20J	20J (Gamma, V3)	C241T,T733C,C2749T,C3037T,C3828T,A5648C,A6319G,A6613G,C12778T,C13860T,C14408T,G17259T,C21614T,C21621A,C21638T,G21974T,G22132T,A22812C,G23012A,A23063T,A23403G,C23525T,C24642T,G25088T,T26149C,G28167A,C28512G,A28877T,G28878C,G28881A,G28882A,G28883C	11288-11296	N:P80R,N:R203K,N:G204R,ORF1a:S1188L,ORF1a:K1795Q,ORF1b:P314L,ORF1b:E1264D,ORF3a:S253P,ORF8:E92K,ORF9b:Q77E,S:L18F,S:T20N,S:P26S,S:D138Y,S:R190S,S:K417T,S:E484K,S:N501Y,S:D614G,S:H655Y,S:T1027I,S:V1176F	ORF1a:S3675-,ORF1a:G3676-,ORF1a:F3677-
Delta	21J.IND	21J (Delta)	G210T,C241T,C3037T,G4181T,C6402T,C7124T,C8986T,G9053T,C10029T,A11201G,A11332G,C14408T,G15451A,C16466T,C19220T,C21618G,G21987A,T22917G,C22995A,A23403G,C23604G,G24410A,C25469T,T26767C,T27638C,C27752T,C27874T,A28461G,G28881T,G28916T,G29402T,G29742T	22029-22034,28248-28253,28271	M:I82T,N:D63G,N:R203M,N:G215C,N:D377Y,ORF1a:A1306S,ORF1a:P2046L,ORF1a:P2287S,ORF1a:V2930L,ORF1a:T3255I,ORF1a:T3646A,ORF1b:P314L,ORF1b:G662S,ORF1b:P1000L,ORF1b:A1918V,ORF3a:S26L,ORF7a:V82A,ORF7a:T120I,ORF7b:T40I,ORF9b:T60A,S:T19R,S:G142D,S:R158G,S:L452R,S:T478K,S:D614G,S:P681R,S:D950N	ORF8:D119-,ORF8:F120-,S:E156-,S:F157-
Omicron	21K (BA.1)	21K (Omicron)	C241T,A2832G,C3037T,T5386G,G8393A,C10029T,C10449A,A11537G,T13195C,C14408T,C15240T,A18163G,C21762T,C21846T,G22578A,T22673C,C22674T,T22679C,C22686T,G22813T,T22882G,G22898A,G22992A,C22995A,A23013C,A23040G,G23048A,A23055G,A23063T,T23075C,C23202A,A23403G,C23525T,T23599G,C23604A,C23854A,G23948T,C24130A,A24424T,T24469A,C24503T,C25000T,C25584T,C26270T,A26530G,C26577G,G26709A,A27259C,C27807T,A28271T,C28311T,G28881A,G28882A,G28883C	6513-6515,11285-11293,21765-21770,21987-21995,22194-22196,28362-28370	E:T9I,M:D3G,M:Q19E,M:A63T,N:P13L,N:R203K,N:G204R,ORF1a:K856R,ORF1a:L2084I,ORF1a:A2710T,ORF1a:T3255I,ORF1a:P3395H,ORF1a:I3758V,ORF1b:P314L,ORF1b:I1566V,ORF9b:P10S,S:A67V,S:T95I,S:Y145D,S:L212I,S:G339D,S:S371L,S:S373P,S:S375F,S:K417N,S:N440K,S:G446S,S:S477N,S:T478K,S:E484A,S:Q493R,S:G496S,S:Q498R,S:N501Y,S:Y505H,S:T547K,S:D614G,S:H655Y,S:N679K,S:P681H,S:N764K,S:D796Y,S:N856K,S:Q954H,S:N969K,S:L981F	N:E31-,N:R32-,N:S33-,ORF1a:S2083-,ORF1a:L3674-,ORF1a:S3675-,ORF1a:G3676-,ORF9b:E27-,ORF9b:N28-,ORF9b:A29-,S:H69-,S:V70-,S:G142-,S:V143-,S:Y144-,S:N211-
